# What Do Experienced Water Managers Think of Water Resources of Our Nation and Its Management Infrastructure?

**DOI:** 10.1371/journal.pone.0142073

**Published:** 2015-11-06

**Authors:** Faisal Hossain, Jeffrey Arnold, Ed Beighley, Casey Brown, Steve Burian, Ji Chen, Anindita Mitra, Dev Niyogi, Roger Pielke, Vincent Tidwell, Dave Wegner

**Affiliations:** 1 Department of Civil and Environmental Engineering, University of Washington, More Hall 201, Seattle, Washington, 98195, United States of America; 2 US Army Corps of Engineers, Institute of Water Resources, Seattle, Washington, 9815, United States of America; 3 Department of Civil and Environmental Engineering, Northeastern University, 360 Huntington Avenue Boston, Masschusetts, 02115, United States of America; 4 Department of Civil and Environmental Engineering, University of Massachusetts Amherst, 130 Natural Resources Road, Amherst, Massachusetts, 01003, United States of America; 5 University of Utah, Department of Civil and Environmental Engineering, 110 Central Campus Drive, Ste 2044, Salt Lake City, Utah, 84112, United States of America; 6 Department of Civil Engineering, Pokfulam Road, The University of Hong Kong, Hong Kong, People’s Republic of China; 7 CREÄ Affiliates, 2319 N 45th Street, Seattle, Washington, 98103, United States of America; 8 Earth and Atmospheric Sciences, Purdue University, 550 Stadium Mall Drive, West Lafayette, Indiana, 47907, United States of America; 9 University of Colorado, CIRES, Boulder, Colorado, 80309–0216, United States of America; 10 Sandia National Laboratories, Albuquerque, New Mexico, 87185, United States of America; 11 U.S. House of Representatives, Washington, DC, United States of America; University California Los Angeles, UNITED STATES

## Abstract

This article represents the second report by an ASCE Task Committee “*Infrastructure Impacts of Landscape-driven Weather Change*” under the ASCE Watershed Management Technical Committee and the ASCE Hydroclimate Technical Committee. Herein, the ‘infrastructure impacts” are referred to as infrastructure-sensitive changes in weather and climate patterns (extremes and non-extremes) that are modulated, among other factors, by changes in landscape, land use and land cover change. In this first report, the article argued for explicitly considering the well-established feedbacks triggered by infrastructure systems to the land-atmosphere system via landscape change. In this report by the ASCE Task Committee (TC), we present the results of this ASCE TC’s survey of a cross section of experienced water managers using a set of carefully crafted questions. These questions covered water resources management, infrastructure resiliency and recommendations for inclusion in education and curriculum. We describe here the specifics of the survey and the results obtained in the form of statistical averages on the ‘perception’ of these managers. Finally, we discuss what these ‘perception’ averages may indicate to the ASCE TC and community as a whole for stewardship of the civil engineering profession. The survey and the responses gathered are not exhaustive nor do they represent the ASCE-endorsed viewpoint. However, the survey provides a critical first step to developing the framework of a research and education plan for ASCE. Given the Water Resources Reform and Development Act passed in 2014, we must now take into account the perceived concerns of the water management community.

## Introduction

Today, water infrastructure is critical to vital sectors of the economy such as energy, transportation, food, and health. These infrastructures comprise dams, levees, irrigation systems, water treatment plants, water retention swales and ponds, potable water supply network and tanks, and manmade as well as natural aquifers, reservoirs, among many. Realizing the importance of water infrastructures, efforts have already begun on understanding the resilience of these systems under a changing climate due to planetary scale global warming using climate projection data. The American Society of Civil Engineers (ASCE) recently set up a Task Committee (TC) titled *“Infrastructure Impacts of Landscape-driven Weather Change”* in 2014 to create a broader understanding of the resilience of water infrastructure in the face of drivers of change. These drivers, such as the local-regional human drivers of landscape change, provide a complementary view to the more well-known green-house-gas (GHG)-based planetary warming as they focus more on mesoscale-to-regional changes that are of more interest to engineering design and operations. Herein, the ‘infrastructure impacts” are referred to as infrastructure-sensitive changes in weather and climate patterns (extremes and non-extremes) that are modulated, among other drivers, by changes in landscape, land use and land cover change. This ASCE TC has summarized its findings from a review of literature on engineering implications for management of water resources in a recent report [1].

In an effort to further the upkeep of water infrastructure for more robust management, a key aspect that often gets ignored is ‘*what do experienced water managers think of the changing patterns water resources*?’ We have already witnessed many scientific studies at local-regional-global scales on recorded and predicted patterns of water resources in the last and current century. For example, studies by [2] have quantified the impact of water regulation on surface water residence time in impounded river basins. [3] have also explored how water resources availability during current and future scenarios may evolve under the pressures of population growth when juxtaposed with projected climate change. Their studies makes a profound conclusion, which is *“(When) climate change is superimposed on the complex hydrologic landscape*, *its signal is difficult to isolate and its influence felt throughout the water supply*, *demand and buffering system”* [3].

More recently, [4] have documented plans that many nations have undertaken in building hydropower dams while others like [5] have explored the impact of current and future water regulation on ecosystem function. These studies provide little insight on the perceptions of experienced water managers at water installations about the state of current and future water resources.

We stress herein the keyword ‘perception’–which is a qualitative yet insightful measure if used judiciously. A qualitative measure does not provide the quantitative rigor needed for the foundations of an engineering design or practice. However understanding the ‘perception’ of experienced water managers and practitioners with decades of water infrastructure management experience can expose issues that need prioritizing. This is especially important given the “*Water Resources Reform and Development Act”* (WRRDA) that was passed in 2014 by United States Congress. While the 2014 Act is quite comprehensive, WRRDA has several aspects that are worth addressing for the engineering community. For example, regarding “River Basins and Coasts areas,” a section of WRRDA:

*(Sec*. *4002) Directs the Secretary of the Army*, *in consultation with specified federal officials*, *to improve forecasting on the Mississippi River by*: *(1) updating forecasting technology deployed on the Mississippi River and its tributaries*, *(2) constructing additional sedimentation ranges on the River*, *and (3) deploying additional automatic identification system base stations at river gage sites*. *Requires the Secretary to report to Congress on activities to improve forecasting and make such report publicly available*.

Similarly on the topic of ‘Water Resources Infrastructure’ of WRRDA, the following is stated:

*(Sec*. *7002) Authorizes the Secretary to carry out final feasibility studies with respect to*: *(1) navigation in Louisiana*, *Florida*, *Georgia*, *Massachusetts*, *and Texas; (2) flood risk management in California*, *Kansas*, *Kentucky*, *Iowa*, *Minnesota*, *Missouri*, *Nevada*, *and North Dakota; (3) hurricane and storm damage risk reduction in California*, *Florida*, *Louisiana*, *and North Carolina; (4) hurricane and storm damage risk reduction and environmental restoration in Mississippi; and (5) environmental restoration in Florida*, *Louisiana*, *Maryland*, *Minnesota*, *North Carolina*, *Oregon*, *and Virginia*.

In this paper, we present the results of this ASCE TC’s survey of a cross section of experienced water managers using a set of carefully crafted questions. These questions covered water resources management, infrastructure resiliency and recommendations for inclusion in education and curriculum. The survey plan reviewed and approved by an institutional review board of American Society of Civil Engineers (ASCE) before the study began. We describe here the specifics of the survey and the results obtained in the form of statistical averages on the ‘perception’ of these managers. Finally, we discuss what these ‘perception’ averages may indicate to the ASCE TC and community as a whole for stewardship of the civil engineering profession.

## Methodology: The Survey

Thirteen key questions were crafted through iterative discussions by the TC. It was felt that a short survey would be appropriate and encourage a quick response. Most of the questions were designed as multiple-choice with the exception of a few that allowed respondents to provide their feedback. Below we outline the questions with multiple-choices. Appendix 1 provides the detailed results of each of these 13 questions. The survey was conducted on www.surveymonkey.com using an ASCE account under the Water Management Technical Council (WMTC). When respondents were sent the link for the survey, they were greeted with an introductory note that explained how the survey results would be used for providing stewardship for the civil engineering profession. This accompanying preamble read as follows:

*“This survey is being carried out under the auspices of the American Society of Civil Engineers (ASCE) by the ASCE Task Committee titled “Infrastructure Impacts of Landscape-Driven Weather Change” under the Watershed Management Technical Council (WMTC)*. *The goal of this survey is to poll practitioners and water managers who have been responsible for the management of water resources and infrastructure over a considerable period of time*. *The survey aims to elicit input on the key issues of observed change in and perception of the distribution of water as a resource due to its management that need to be addressed for future policy and planning in the 21* ^*st*^ *century*. *The data gathered from this survey will initiate a dialogue among various stakeholders (academia*, *legislative bodies*, *practitioners and public) that is needed to ensure alignment of goals and ensure a more sustainable and resilient water management infrastructure*. *The data gathered will also guide the ASCE Task Committee on the future work that needs to be carried out to further the understanding of water infrastructure impacts of landscape-driven weather change*. *This issue has traditionally not been at the forefront of engineering practice*. *Thus*, *completion of the survey will provide strategic guidance to help the committee produce publications*, *reports*, *webinars and guiding documents for positively impacting the engineering practice for future generations*.*”*

**Question 1:** Over the last few decades, what would you rate as the most significant change in the distribution of water resources (surface or ground) in the area in which you have managed or worked?

- Change in magnitude of extremes (low flows or high flows)?
- Change in frequency of extremes (low flows or high flows)?
- Temporal trend in extremes (declining or rising trend)?
- Change in variability (compared to mean flow)?
- Other (elaborate in a few words below)

Upload your document (optional)

**Question 2:** In your opinion, what are the likely external drivers of change in the distribution of water resources that you indicated in Question 1? [Note: You may select more than one.]

- None. It’s all within the natural range of variability
- Terrestrial–Land use/land cover (landscape) change resulting in change in infiltration patterns (e.g. increasing imperviousness).
- Terrestrial–land-atmosphere feedbacks [landscape driven changes in weather and climate]
- Atmospheric–Changing weather patterns from natural and/or human effects
- Engineering–Human management and redistribution of water resources (e.g. diversions, impoundments, irrigation projects; inter-basin transfer; inadequate storm water management)
- Other–please name it below.

Upload your document (optional)

**Question 3:** If you selected more than one answer in Question 1, please rank your responses from most significant to least significant (for example, b, d, e).

**Question 4:** Do you feel the water quality of water resources (e.g. both surface and ground) has experienced any significant change over the last few decades due to landscape change?

- Yes
- No

**Question 5:** If you answered yes to Question 4, what do you believe could be the key reason for the change in water quality?

- Non-point source pollution (e.g. fertilizer application; agricultural practice; industrial waste)
- Urbanization
- Atmospheric processes (e.g. acidification; aerosol)
- Other–please name it below.

Upload your document (optional)

**Question 6:** What are the most concerning implications and impacts of the aforementioned changes on water management and associated infrastructure?

**Question 7:** Do you feel the conventional techniques and management practices used for managing water resources by large water infrastructures are currently adequate for the 21^st^ century?

- Yes
- No
- N/A

Upload your document (optional)

**Question 8:** If you had to prioritize areas of research required for improving water management using infrastructure for the 21st century, what would they be?

- Understanding engineering implications on distribution of water resources;
- Developing flexible operational procedures for water management;
- Conducting risk management and vulnerability assessments for water infrastructure;
- Understanding the uncertainty of the role of external drivers (such as climate change);
- Other–please elaborate briefly

Upload your document (optional)

**Question 9:** Please outline briefly any knowledge gaps in external drivers (landscape or weather) that you feel currently prevents the engineering community from formulating practical solutions for more resilient water infrastructure (Note: These gaps may be considered as ‘recommended research areas’).

**Question 10:** In follow up to Question 9, what specific type of information or assessment do you think is most useful in immediately and positively impacting engineering practices? (Please limit your response to 250 characters)

**Question 11:** Do you feel the current curriculum in environmental engineering or in the sciences is adequate to inform a graduate (B.S. degree) of the challenges facing water resource management this century?

- Yes
- No
- No opinion

**Question 12:** If you answered No to Question 11 above, please elaborate briefly on the type of curriculum changes needed (e.g. an undergraduate course on the human impacts of weather/climate, surface hydrology; water management; hydrometeorology; land management).

**Question 13** (Last Question!): Please provide some information about your profession:

- State or region with which you are most concerned
- Affiliation (academia; think tank, federal/state agency, non-profit)

### Profile of Respondents

**Fig 1:**
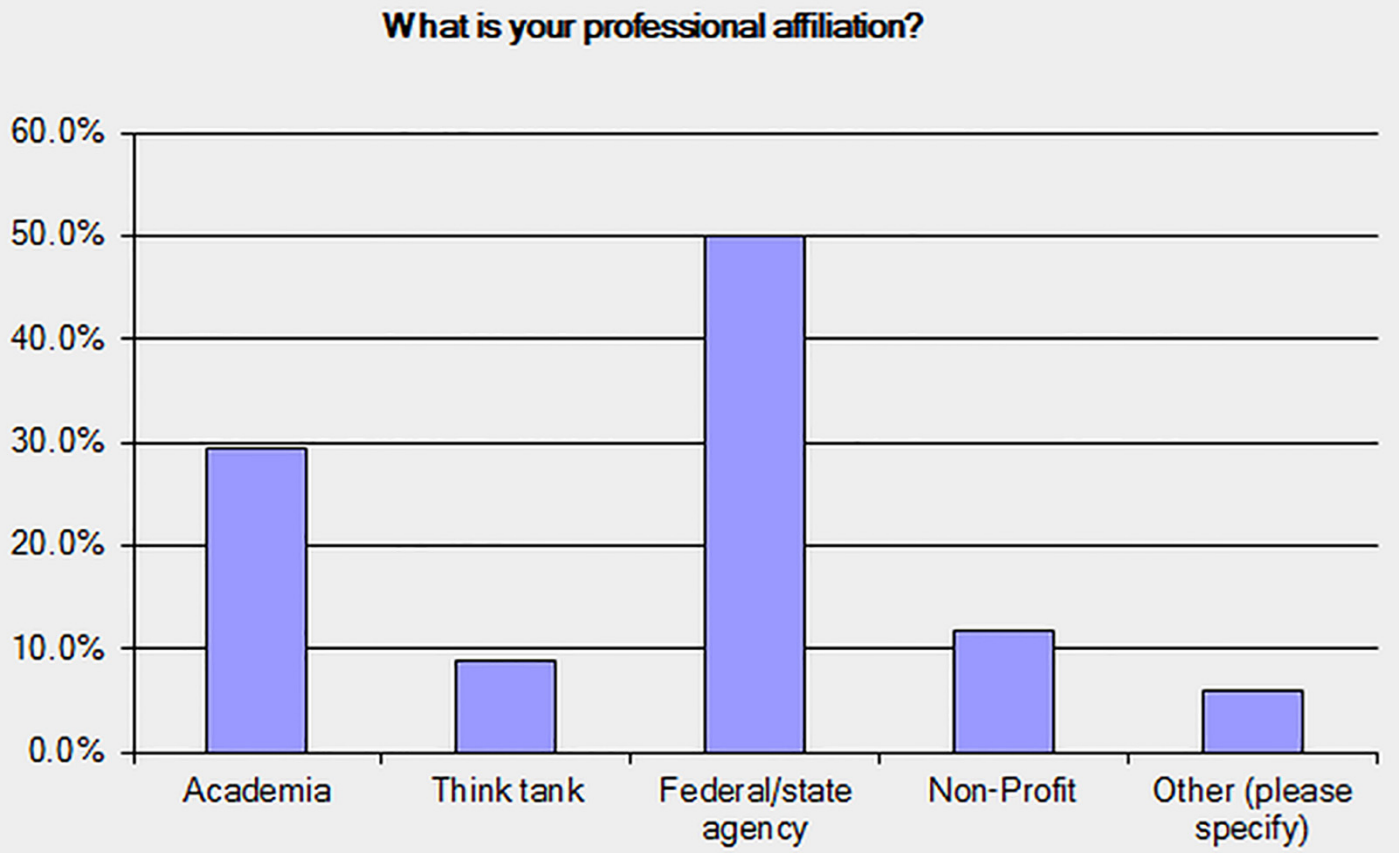
Profile distribution of respondents for the ASCE TC survey on perceptions of water resources.

The survey was sent out to 90 potential respondents who met the following qualifiers: 1) operational involvement with water management and/or water infrastructure facility/installation 2) Two or more decades of experience in water management and decision making 3) practitioner, researcher or academician, or think-tank member. Those surveyed included numerous water facility managers of federally owned facilities as well as city (metro) facilities, state climatologists, and private consulting (for-profit) firms in the water sector. Anonymity was guaranteed. The survey was released on February 5, 2015 and closed after a 4-month period punctuated with periodic reminders. After several repeated reminders, 44 respondents provided their responses, which the TC considered adequate as a fair representation of this group. Fig 1 summarizes the relative distribution of respondent type by sector, with the majority from Federal/state agencies.

### Discussion of Survey Results

Summary of survey results in the form of graphs are shown in Appendix 1. On the first question, most respondents opined that change in frequency of low or high flows was becoming more noticeable than the change in flow values per se (Figures shown in Appendix 1). Thus, it seems that water managers are feeling the extremely high or low flow values as becoming either more frequent or less frequent than before. One can argue that this may potentially be an effect of the increasing regulation, damming and diversion/withdrawal of freshwater where outflow systems are often operated episodically based on societal demand. Majority of respondents feel the changes are driven by atmospheric drivers (natural and human-modified weather patterns). However, the interesting nuance to this issue (question 2; Fig A in S1 Appendix) is that respondents also believe that landscape change that result in changing infiltration patterns (due to altered perviousness) is another likely driver of the change in water resources patterns. An overwhelming 70% believe that water quality has been impacted over the last few decades just as much as water quantity (Fig B in S1 Appendix). This change in water quality is attributed to two dominant drivers–non point source pollution (41%) and urbanization (27.6%) (Fig E in S1 Appendix). What is clear from this particular response is that future assessment of water availability should encompass both quality and quantity (flux and volume) metrics in a coordinated manner that is mostly absent today. Without one the value of other is harder to define.

There was a broad perspective of *the most concerning implications and impacts of the changes on water management and associated infrastructure discussed above*. Some of the most unique or common responses are listed below:

1. If more extreme events (major floods our droughts) are becoming more common, our assumptions of stationarity must be replaced with something else that can be easily used/understood by practitioners in the field.
2. Lack of regulation on nonpoint source pollution
3. In the West land use has a direct impact on water availability
4. Historical distributions may not be representative of evolving distribution of extreme events
5. Uncertainty associated with links between urbanization and its impacts
6. Reduced reliability (due to less certainty @ underlying distribution) of water supplies.
7. Future increases in population will accelerate decrease in quality.
8. Application of nutrients, particularly animal waste on agricultural lands.
9. Rural water (small town) availability from confined aquifers over appropriation of irrigation wells within Natural Resource Districts.
10. First most urban infrastructure is not currently built to treat for all of these contaminants and that coupled with salinity increases will drive the need to build new enhanced treatment facilities that will be very expensive. These newer facilities will in all likelihood also drive an increase in power consumption. Hence urban water rates are going to experience significant increases.
11. Too much emphasis is placed upon short-term (few decades) history, longer-term data (100–150 years) shows wide range of extremes/short-term trends that many scientists seem to be unaware of and have become too dependent upon personal short-term perception and less from the full suite of historical data available.
12. Recognizing the mix between urban and rural pollution sources with the role that urbanization has in these processes.

The key concerns of most water managers appear to be

- reduced reliability of water delivery systems;
- role of rural systems in relation to ever expanding and resource-hungry urban systems; and
- rising costs for addressing emerging contaminants in water that will make water systems more expensive.

Given these concerns, an overwhelming (71%) of respondents do not feel that conventional techniques and management practices used for managing water resources are adequate for the 21st century (Question 7; Fig F in S1 Appendix). As a potential solution, most respondents feel that it is worthwhile to make current operational protocols more flexible (62%) while accounting for the uncertainty in the drivers of change in water resources (41%) (Fig G in S1 Appendix).

In terms of knowledge gaps that need to be addressed for better management of our water resources and infrastructure (Question 9; Fig H in S1 Appendix), key responses included the following:

1. Understanding the range of uncertainty in hydrologic climate change impacts
2. Helping decision makers understand the implications of various solutions, including the do nothing approach.
3. Feedback between land use changes and atmospheric reactions.
4. The role of land drainage modifications (such as tile drains) on streamflow.
5. Development of improved historical environmental data bases for precipitation, temperature, streamflow, snow cover, etc. Also much better understanding (through field trials, not modeling) of the hydrologic implications of land use practices (tillage methods, crop types, soil erosion, drainage practices, etc.
6. Impacts from droughts, wildfires, climate change. For example, I've seen knowledge gaps in drinking water infrastructure and have become more visible during drought.
7. Need to learn how to take the systems approach better.
8. I think the gaps are in political consensus and political will. The water management community should formulate workable solutions and communicate to increase their practical implement ability.
9. Lack of knowledge about implications of climate change on variability of local-to-regional scale precipitation patterns.

On the question of required changes to undergraduate curriculum for training the future generation, a general sense of ambivalence was noted. More than half of the respondents responded ‘*No Opinion*’ indicating that most water managers have probably not given much thought to how the higher education model for engineers may need to be adjusted to suit the emerging needs of water management. This perhaps points to the need for greater collaboration between academics and practicing water managers in designing use-inspired curriculum that addresses new challenges. Finally, when asked about the region within United States that respondents were most concerned about for water management, an overwhelming majority cited the Western US. This endorses the viewpoint that the Western US is in greater need of adaptive and creative solutions to manage its increasingly scarce water resources than the rest of the country.

## Conclusion

It is important to stress that the survey results do not necessarily reflect a view that is endorsed by ASCE or any particular agency. However, the survey of the perceptions of experienced water managers revealed several insights that the engineering community should take heed of as it formulates better management strategies for the 21^st^ century. The wide ranging viewpoints provide a catalyst for discussions on catalyzing changes. For example, it is clear that much thought has not been given on the required academic-practitioner collaboration in developing more use-inspired curriculum for our future engineers who will manage our water infrastructure of tomorrow. Similarly, water quality issues can no longer be treated separate from water resource management issues that focus mostly on quantity. The very essence of a resource implies both quality and quantity; without one the other is quite hard to define. In this regard, the emergence of new contaminants or changes to quality due to varying quantity brought by climate variability such as drought or flood will likely add to the cost of water delivery systems–an issue that is not yet in the limelight. Lastly, engineers concede that understanding the uncertainty brought about by future climate variability on water is as a critical knowledge gap hampering effective adaptive practices. Engineers have never avoided uncertainty and have always found a durable way to address it in design and operations. As we try to figure out how engineering practices should adapt to make our water infrastructure more resilient, quantification of this future uncertainty will be important.

The survey and the responses gathered are not exhaustive. They provide a critical first step to developing the framework of a research and education plan for ASCE. Given the Water Resources Reform and Development Act passed in 2014, we must now take into account the perceived concerns of the water management community.

## Supporting Information

S1 Appendix Survey Results. (DOCX)

S1 File Survey Data on questions and raw (unedited) responses from respondents. (XLS)

S2 File Survey Data summarized as charts and statistics. (PDF)
