# Supplementary material for: What Do Experienced Water Managers Think of Water Resources of Our Nation and Its Management Infrastructure?
Source: PLoS One. 2015 Nov 6;10(11):e0142073. doi: 10.1371/journal.pone.0142073 (PMC4636134; doi:10.1371/journal.pone.0142073)
Supplement: S1 Appendix — (DOCX) [file pone.0142073.s001.docx]

**APPENDIX 1: SURVEY RESULTS**

**Fig A in S1 Appendix**

**Fig B in S1 Appendix**

**Fig C in S1 Appendix**

**Fig D in S1 Appendix**

**Fig E in S1 Appendix**

**Fig F in S1 Appendix**

**Fig G in S1 Appendix**

**Fig H in S1 Appendix**
