# Supplementary material for: What Do Experienced Water Managers Think of Water Resources of Our Nation and Its Management Infrastructure?
Source: PLoS One. 2015 Nov 6;10(11):e0142073. doi: 10.1371/journal.pone.0142073 (PMC4636134; doi:10.1371/journal.pone.0142073)
Supplement: S2 File — (PDF) [file pone.0142073.s003.pdf]

Q1 Over the last few decades, what would you rate as the most significant change in the distribution of water resources (surface or ground) in the area in which you have managed or worked?

Answered: 44 Skipped: 0

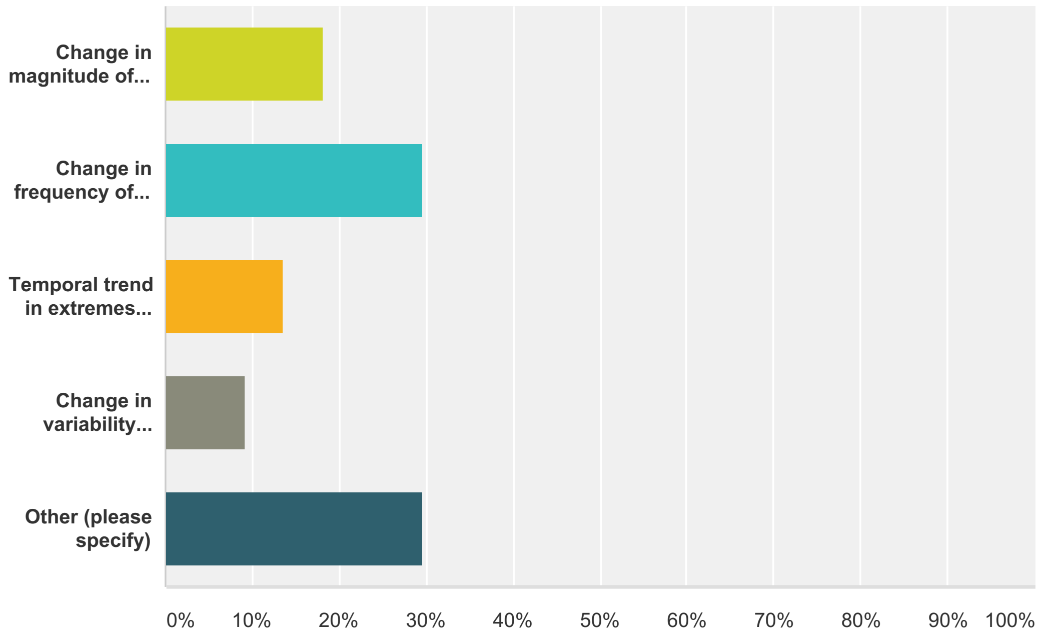

Answer Choices	Responses	
Change in magnitude of extremes (low flows or high flows)	**18.18%**	8
Change in frequency of extremes (low flows or high flows)	**29.55%**	13
Temporal trend in extremes (declining or rising trend)	**13.64%**	6
Change in variability (compared to mean flow)	**9.09%**	4
Other (please specify)	**29.55%**	13
**Total**		**44**

#	Other (please specify)	Date
1	This really depends on the region of the country we are talking about. But I would say that change in magnitude and frequencies are the top two.	4/30/2015 5:02 PM
2	It seems that high flows may be occurring more frequently	3/31/2015 4:46 PM
3	It is both the magnitude of the extremes and the frequency	3/24/2015 3:17 PM
4	no change	3/23/2015 10:11 AM
5	In general, it is very hard to separate climate variability and climate change in what has happened over the course of my professional career. Two things stand out 1) declines in groundwater, which are certainly a result of human activity, and 2) shifting seasonality - a particular problem in snow dependent systems like California, and which are likely influenced by human activity.	3/23/2015 9:39 AM

6	Change in Magnitude of Extremes, AND, change in timing of those extremes (due to changing weather patterns - e.g. earlier and higher peak flows off the sierra nevada due to incresaed temps and rain on snow events)	3/23/2015 8:56 AM
7	no changes	3/23/2015 8:15 AM
8	Apparent manifestation of being in a lower cycle end of a decadal scale variability that is present in historical (including paleo) records.	3/16/2015 5:47 PM
9	Land use changes	3/12/2015 11:14 AM
10	Change in mean	2/17/2015 4:46 PM
11	From my perspective, the most significant change is a HEIGHTENED AWARENESS of our uncertainty about magnitude and frequency of extremes and the need to plan, design, build, and operate with an appreciation of that.	2/17/2015 12:29 PM
12	Now change - as a State Climatologist I monitor these variations with real data.	2/16/2015 9:08 AM
13	In Colorado, you could check all four top boxes if you believed in this, but for many gages we have short records. We need more data to give answers to these questions.	2/14/2015 12:31 PM

Q2 In your opinion, what are the likely external drivers of change in the distribution of water resources that you indicated in Question 1? [Note: You may select more than one.]

Answered: 44   Skipped: 0

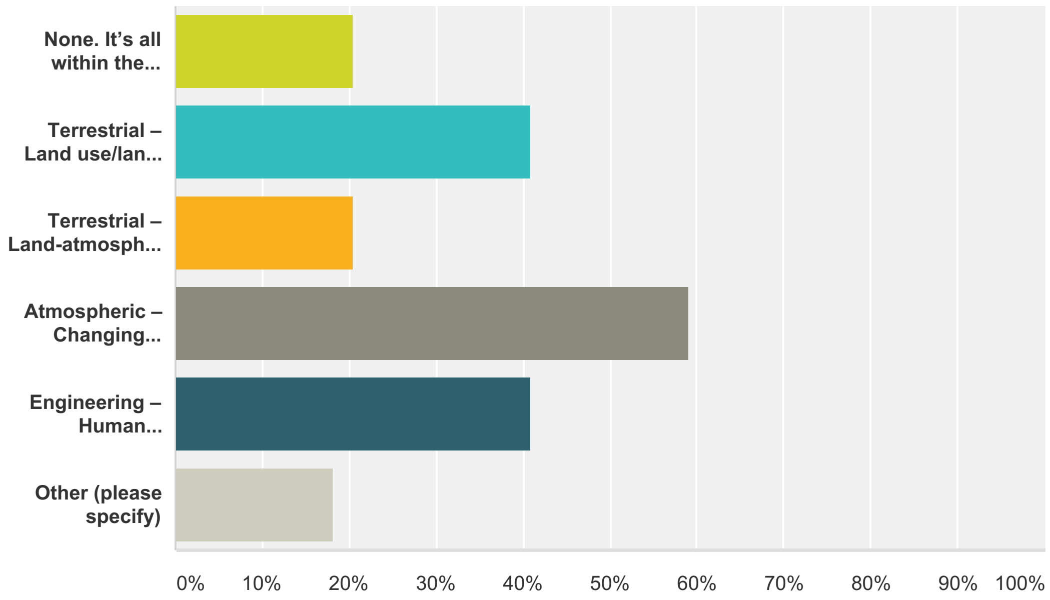

Answer Choices	Responses	
None. It's all within the natural range of variability	**20.45%**	9
Terrestrial – Land use/land cover (landscape) change resulting in change in infiltration patterns	**40.91%**	18
Terrestrial – Land-atmosphere feedbacks [landscape driven changes in weather and climate]	**20.45%**	9
Atmospheric – Changing weather patterns from natural and/or human effects	**59.09%**	26
Engineering – Human management and redistribution of water resources	**40.91%**	18
Other (please specify)	**18.18%**	8
**Total Respondents: 44**		

#	Other (please specify)	Date
1	Drivers of change are not my field of research and not relevant to my work.	3/23/2015 10:53 AM
2	All of these things have occurred in one or more of the basins I've worked in. The third is the most difficult to document. You do not mention declining flows due to declining groundwater, nor do you mention sea level rise. They deserve their own categories, in my opinion.	3/23/2015 9:39 AM
3	Its a combination of all of the above.	3/17/2015 5:43 PM
4	While I may have personal opinions about this, those don't really matter in our work as consultants to water agencies. The causes are not as important as the outcomes and our ability to deal for them.	2/17/2015 12:29 PM

5	The changes in extremes I've observed have been attributed by the science community as being due to natural variability. However, they occur under conditions (e.g., warmest decade in historical record) that are very likely attributable to climate change. These background conditions exacerbate the impacts of the extremes -- for example, further reducing snowpack in a drought year.	2/17/2015 12:12 PM
6	Policy - the rising control exerted by a centralized government on water resources will change the way the resources are managed, utilized and distributed, generally for the detriment of economic development.	2/16/2015 9:08 AM
7	I don't think I can provide an intelligent answer to this question. It almost seems like a leap of faith rather than something based on data.	2/14/2015 12:31 PM
8	In reality it is a mix of all things above. It is difficult to separate individual effects	2/14/2015 6:35 AM

Q3 If you selected more than one answer in Question 1, please rank your responses from most significant to least significant (for example, b, d, e).

Answered: 25 Skipped: 19

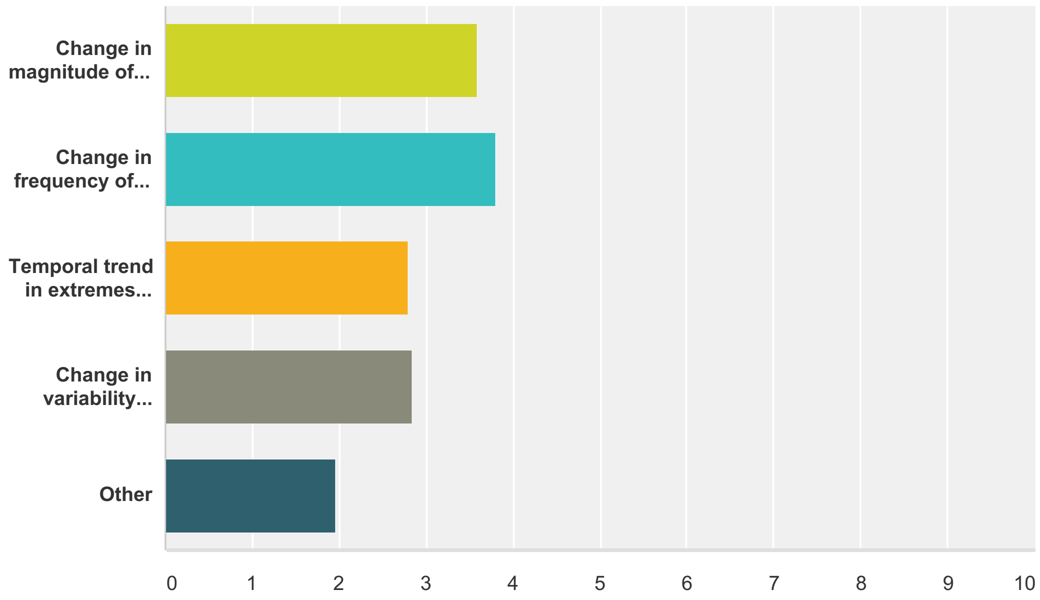

	1	2	3	4	5	Total	Score
Change in magnitude of extremes (low flows or high flows)	**24.00%** 6	**32.00%** 8	**24.00%** 6	**20.00%** 5	**0.00%** 0	25	3.60
Change in frequency of extremes (low flows or high flows)	**40.00%** 10	**16.00%** 4	**32.00%** 8	**8.00%** 2	**4.00%** 1	25	3.80
Temporal trend in extremes (declining or rising trend)	**8.00%** 2	**20.00%** 5	**24.00%** 6	**40.00%** 10	**8.00%** 2	25	2.80
Change in variability (compared to mean flow)	**4.00%** 1	**32.00%** 8	**20.00%** 5	**32.00%** 8	**12.00%** 3	25	2.84
Other	**24.00%** 6	**0.00%** 0	**0.00%** 0	**0.00%** 0	**76.00%** 19	25	1.96

Q4 Do you feel the water quality of water resources (e.g. both surface and ground) has experienced any significant change over the last few decades due to landscape change?

Answered: 43 Skipped: 1

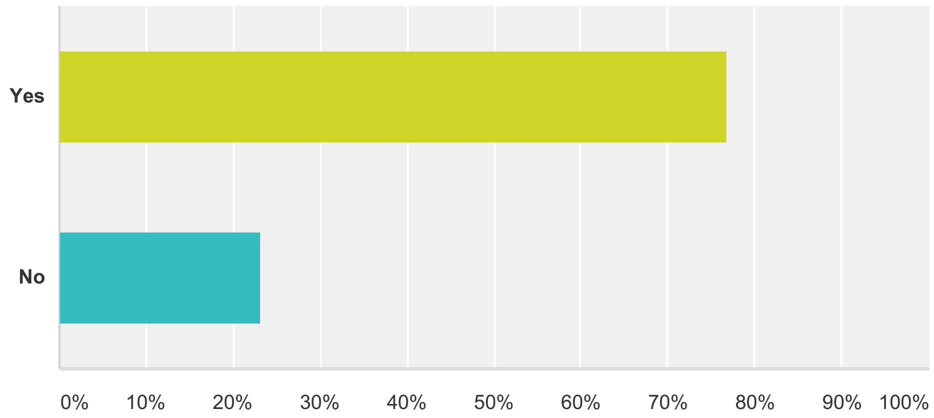

Answer Choices	Responses	
Yes	76.74%	33
No	23.26%	10
Total		43

Q5 What do you believe could be the key reason for the change in water quality?

Answered: 33 Skipped: 11

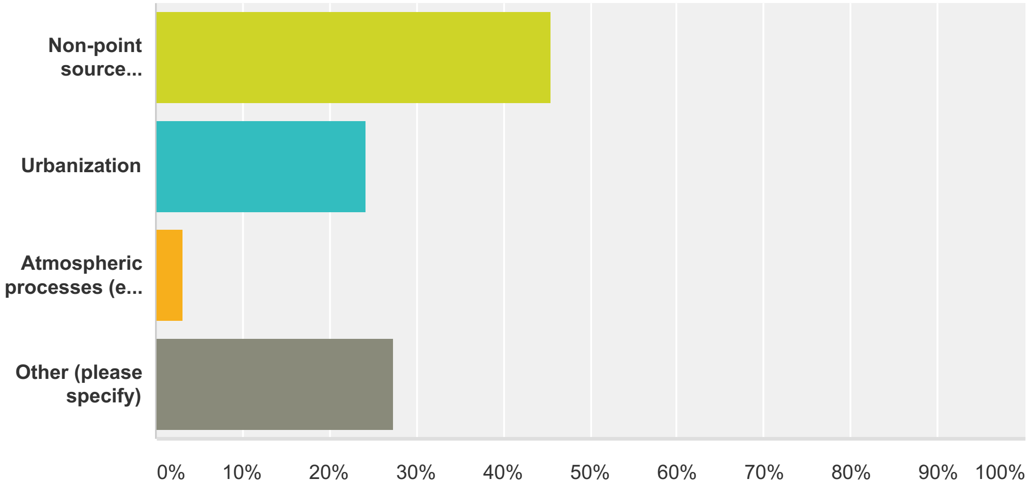

Answer Choices	Responses	
Non-point source pollution (e.g. fertilizer application; agricultural practice; industrial waste)	**45.45%**	15
Urbanization	**24.24%**	8
Atmospheric processes (e.g. acidification; aerosol)	**3.03%**	1
Other (please specify)	**27.27%**	9
**Total**		**33**

#	Other (please specify)	Date
1	Urbanization, non-point source pollution and climatechange continue to play an impartant role in water quality degradation.	5/5/2015 3:13 PM
2	All of the last 3 questions are very basin specific. Having worked in many basins, I am of the opinion that generic answers to these 3 questions are NOT useful. The list of potential issues is useful in evaluating a particular basin, but the answers for that basin are highly likely to be inappropriate for other basins. In other words, I would place little significance on the answers to the last three questions. I have seen 1 or more of these issues arise in many of the basins I've worked on.	3/23/2015 9:57 AM
3	This is a push question. Overall quality is better in many places over the past few decades.	3/23/2015 8:12 AM
4	All of the above	3/17/2015 5:46 PM
5	Warmer air temperatures have led to higher summer temperature water flows which combined with the more frequent below average precipitation/runoff have caused elevated streamflow temperatures	3/16/2015 5:54 PM
6	In the Southwest, decreasing reservoir levels have caused a sharp increase in salinity and concentration of contaminants.	2/17/2015 5:10 PM
7	drought - wildfire	2/17/2015 2:52 AM
8	All of above here	2/14/2015 12:33 PM
9	Combination of agricultural practices in some areas and mining operations in other areas	2/13/2015 10:22 PM

Q6 What are the most concerning implications and impacts of the aforementioned changes on water management and associated infrastructure?

Answered: 42 Skipped: 2

#	Responses	Date
1	x	5/8/2015 10:08 AM
2	Our current infrastructure is not designed to handle the extremes we are facing. Addtionally, again water infrastructure is a big concern.	5/5/2015 3:13 PM
3	Cyanotoxins will be a major impact moving forward. There are many, many other issues to be concerned about.	5/1/2015 9:24 PM
4	Design standards may not account for these changes, so adaptations, future thought in how to design systems is needed.	5/1/2015 10:02 AM
5	We've already experienced some of the impacts in terms of the Gulf of Mexico and polluted ground water	4/30/2015 4:41 PM
6	If more extreme events (major floods our droughts) are becoming more common, our assumptions of stationarity must be replaced with something else that can be easily used/understood by practitioners in the field.	3/31/2015 4:51 PM
7	lack of regulation on nonpoint source pollution	3/30/2015 10:00 PM
8	In the West land use has a direct impact on water availability	3/24/2015 3:27 PM
9	Historical distributions may not be representative of evolving distribution of extreme events	3/23/2015 12:19 PM
10	Uncertainty associated with links between urbanization and its impacts	3/23/2015 10:57 AM
11	urbanization	3/23/2015 10:13 AM
12	In no particular order: Utility of supply, environmental impacts on instream and riparian habitat, impact on commercial fisheries, cost of treatment.	3/23/2015 9:57 AM
13	Reduced reliabliity (due to less certainty @ underlying distribution) of water supplies. Increased disaster recovery spending Less confidence by the public in technical expertise.	3/23/2015 9:02 AM
14	impact on natural resources and functions	3/23/2015 8:32 AM
15	Future increases in population will accelerate decrease in quality.	3/23/2015 8:17 AM
16	Another push question. Implies only negative ignoring gains. Obviously must continue to work and keep ahead of increasing population/urbanization/sprawl, etc.	3/23/2015 8:12 AM
17	point source control is not adequate to offset non-point	3/23/2015 7:43 AM
18	Application of nutrients, particularly animal waste on agricultural lands.	3/23/2015 6:47 AM
19	Shifts in rainfall, finer scale changes beyond what we have traditionally seen/noted/studied	3/17/2015 5:46 PM
20	Increased reliance on water management infrastructure to accomodate changes	3/16/2015 5:54 PM
21	Degraded water quality	3/12/2015 11:21 AM
22	Rural water (small town) availability from confined aquifers over appropriation of irrigation wells within Natural Resource Districts	2/25/2015 10:33 AM
23	First most urban infrastructure is not currently built to treat for all of these contaminants and that coupled with salinity increases will drive the need to build new enhanced treatment facilities that will be very expensive. These newer facilities will in all likelihood also drive an increase in power consumption. Hence urban water rates are going to experience significant increases.	2/17/2015 5:10 PM
24	N/A	2/17/2015 5:07 PM

25	reduced reliability of base streamflows and resulting water supplies	2/17/2015 4:47 PM
26	perhaps longer term drought than e have previously experienced, but which has occurred based upon tree ring analysis	2/17/2015 3:52 PM
27	its ability to withstand the variability and magnitudes	2/17/2015 1:19 PM
28	Too much emphasis is placed upon short-term (few decades) history, longer-term data (100-150 years shows wide range of extremes/short-term trends that many scientists seem to be unaware of and have become too dependent upon personal short-term perception and less from the full suite of historical data available.	2/17/2015 1:13 PM
29	Greater stress on existing infrastructure given increasing uncertainty due to extreme events, unanticipated growth, and environmental demands.	2/17/2015 12:30 PM
30	Water shortage	2/17/2015 12:16 PM
31	reduced water supply, both surface and ground water	2/17/2015 2:52 AM
32	economy, environment, equity	2/16/2015 3:01 PM
33	The limitation of the use of water resources for sensible economic activity will have the greatest impact - negative impact on people's ability to thrive.	2/16/2015 9:12 AM
34	from a western US perspective, the resources are stretched beyond that of existing water management requirements	2/16/2015 1:12 AM
35	Increased risk of system failure	2/15/2015 1:40 PM
36	A ratcheting effect downward in water quality and e-flow availability	2/14/2015 12:33 PM
37	Eutrophication, loss of oxygen	2/14/2015 12:01 PM
38	Reduced assurance of water supply	2/14/2015 11:26 AM
39	Decreasing reliability, ecosystem damage,	2/14/2015 10:34 AM
40	Recognizing the mix between urban and rural pollution sources with the role that urbanization has in these processes.	2/14/2015 8:31 AM
41	quality of water in receiving water bodies saltwater intrusion and water supply nuisance floodimg	2/14/2015 6:39 AM
42	No major impacts	2/13/2015 10:22 PM

Q7 Do you feel the conventional techniques and management practices used for managing water resources by large water infrastructures are currently adequate for the 21st century?

Answered: 42 Skipped: 2

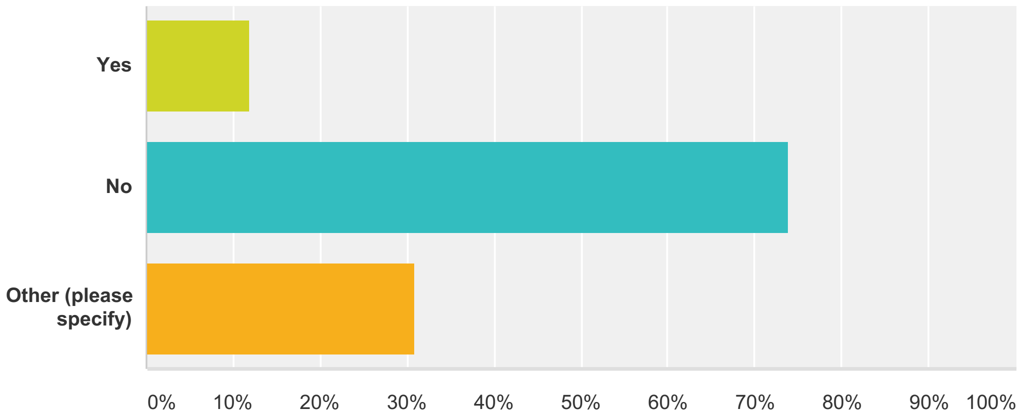

Answer Choices	Responses	
Yes	**11.90%**	5
No	**73.81%**	31
Other (please specify)	**30.95%**	13
**Total Respondents: 42**		

#	Other (please specify)	Date
1	From water utility perspective - adaptive management is the technique that has been applied. That will get us part of the way, but how the practices are regulated (rule curves, policy frameworks, funding mechanisms for infrastructure) may need to be adapted to address these changes. Certainly some of the other standards of practice may need to be changed too.	5/1/2015 10:02 AM
2	Currently adequate for the basins where I have a job responsibility, but need to have flexible operational procedures that can be enacted when needed. This means developing the flexible operational procedures now -- not waiting until a major flood or drought and THEN working to develop procedures.	3/31/2015 4:51 PM
3	Techniques and management practices are constantly changing and improving to meet evolving needs.	3/23/2015 10:57 AM
4	I have made a career of suggesting simple, generally systems based, approaches that greatly enhance the benefits and reduce the costs associated with managing water resources. Current management practices are often based on independent management of the pieces of a system, these are often not efficient, although they may be adequate - Boston water supply is an example where the infrastructure can (NOT saying it is) be inefficiently managed and still be adequately managed. California is a place where efficient management may still be inadequate. Repeating myself, it is hard to generalize about water management.	3/23/2015 9:57 AM
5	Conventional techniques serve as a strong basis. They cannot simply be jettisoned. Instead they need to be revised and supplemented by increased understanding both from a hydrologic/biologic/atmosphereic perspective, _and_ from the social science perspective (via better communication/ public involvement)	3/23/2015 9:02 AM
6	Continuing improvements in addition to current practice will be necessary, particularly in operations management using both demand and supply management techniques.	3/23/2015 8:12 AM

7	Probably not. There is a need for some modification.	3/17/2015 5:46 PM
8	I do feel engineering techniques are probably adequate, but costs will become prohibitive if we continue to have to treat/manage larger and larger volumes of water due to degraded quality and increased volumes of runoff.	3/12/2015 11:21 AM
9	It very much depends on the region you are talking about.	2/17/2015 5:10 PM
10	There is a need for greater flexibility in the operation of large projects and a related need for more data, in real time, as well as improved season and interannual predictive capacity and skill.	2/17/2015 12:30 PM
11	There isn't the political will needed to make the large investments in infrastructure necessary not only to catch up from past decades of neglect but also to build resiliency for the future.	2/17/2015 12:16 PM
12	I believe the huge western projects are environmentally and economically unsustainable. In the Eastern US where water is abundant most of the time there will be greater resilience and more security for those major parts of the economic system that rely on fresh water. I see a migration of irrigated agriculture back to the East where only a fraction of that used in the West is needed.	2/16/2015 9:12 AM
13	The whole issue of nonstationarity needs a new paradigm. What we have in text books do not apply anymore	2/14/2015 6:39 AM

Q8 If you had to prioritize areas of research required for improving water management using infrastructure for the 21st century, what would they be?

Answered: 42 Skipped: 2

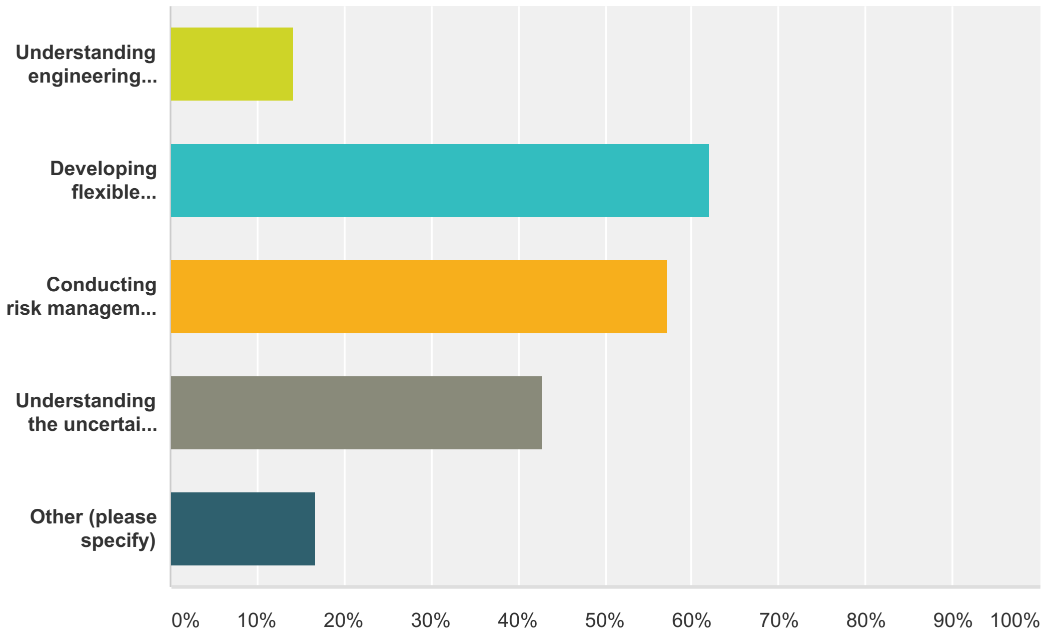

Answer Choices	Responses	
Understanding engineering implications on distribution of water resources;	**14.29%**	6
Developing flexible operational procedures for water management;	**61.90%**	26
Conducting risk management and vulnerability assessments for water infrastructure;	**57.14%**	24
Understanding the uncertainty of the role of external drivers (such as climate change);	**42.86%**	18
Other (please specify)	**16.67%**	7
**Total Respondents: 42**		

#	Other (please specify)	Date
1	Understanding and overcoming human obstacles to better management	3/23/2015 9:57 AM
2	Risk Communication	3/23/2015 9:02 AM
3	Higher resolution analysis	3/17/2015 5:46 PM
4	Understanding land use-water management relationships	3/12/2015 11:21 AM
5	All four are equally important and have to be viewed together.	2/17/2015 5:10 PM
6	Development of low-impact water systems in the East	2/16/2015 9:12 AM
7	Better approach to total water management, including infrastructure	2/14/2015 12:33 PM

Q9 Please outline briefly any knowledge gaps in external drivers (landscape or weather) that you feel currently prevents the engineering community from formulating practical solutions for more resilient water infrastructure (Note: These gaps may be considered as ‘recommended research areas’).

Answered: 34 Skipped: 10

#	Responses	Date
1	x	5/8/2015 10:08 AM
2	Downscaling and uncertainty with the current climate models - it is hard to gain support for a project based on such a large uncertainty.	5/5/2015 3:15 PM
3	I don't understand what this question is asking for.	5/1/2015 9:25 PM
4	uncertainty in projections and wide error bars for changes in the hydrologic cycle	4/30/2015 4:43 PM
5	Understanding the range of uncertainty in hydrologic climate change impacts	3/31/2015 4:54 PM
6	causal mechanisms for extreme events and the changes in those processes in coming decades	3/23/2015 4:49 PM
7	Arrgh!! Depends where you are and the problem you're trying to solve. In undeveloped places, ther may be no hydrologic data. In complex places where efficient management is important, forecasts. Other stuff, too.	3/23/2015 10:02 AM
8	water use trends	3/23/2015 9:07 AM
9	Helping decision makers understand the implications of various solutions, including the do nothing approach	3/23/2015 8:35 AM
10	Effects of changing land use/population/technologies on existing projects and demands/performance expectations. More clarity in understanding long term weather variations and extremes compared to climate.	3/23/2015 8:27 AM
11	feedback between land use changes and atmospheric reactions	3/23/2015 8:19 AM
12	political constraints	3/23/2015 7:44 AM
13	The role of land drainage modifications (such as tile drains) on streamflow.	3/23/2015 6:49 AM
14	soil moisture information, gridded datasets, if- then scenarios and protocols, analogs.	3/17/2015 5:47 PM
15	Monitoring gaps in processes and forecasting products ranging from 2 wks out to 3 months that can result in some type of water resources action	3/16/2015 5:56 PM
16	Importance of proper land use management to improve soil and soil health in order to retain more water on the landscape to prevent having to build infrastructure larger and larger to handle increasing volumes of runoff.	3/12/2015 11:23 AM
17	managed river flow impacts on alluvial aquifer recharge in heavy agricultural irrigation areas The impact of climate on future river flows and deep aquifer recharge	2/25/2015 10:42 AM
18	N/A	2/17/2015 5:08 PM
19	lack of widely accepted techniques for accounting for future uncertainty in climate change	2/17/2015 4:48 PM
20	need better downscaled climate to runoff models	2/17/2015 3:54 PM
21	Development of improved historical environmental data bases for precipitation, temperature, streamflow, snow cover, etc. Also much better understanding (through field trials, not modeling) of the hydrologic implications of land use practices (tillage methods, crop types, soil erosion, drainage practices, etc.	2/17/2015 1:21 PM
22	the ability of infrastructure to withstand extremes in the fuure	2/17/2015 1:20 PM

23	Risk assessment and mitigation given increasing uncertainty related to weather extremes, as well as pressing political/environmental mandates.	2/17/2015 12:37 PM
24	1. Improved seasonal and sub-seasonal precipitation prediction 2. Procedures for flood frequenty analysis that take hydrologic non-stationarity into account	2/17/2015 12:18 PM
25	none	2/17/2015 2:54 AM
26	institutional inertia legislative paranoia	2/16/2015 3:02 PM
27	Understanding the true economic and environmental costs of supporting a western US water system which was enabled by Federal expenditures which also caused economic devastation to large agricultural areas of the East.	2/16/2015 9:15 AM
28	impacts from droughts, wildfires, climate change. For example, I've seen knowledge gaps in drinking water infrastructure and has become more visible during drought	2/16/2015 1:48 AM
29	Need to learn how to take the systems approach better	2/14/2015 12:34 PM
30	I think the gaps are in political consensus and political will. The water management community should formulate workable solutions and communicate to increase their practical implementability.	2/14/2015 11:31 AM
31	More regionally informative climate models	2/14/2015 10:35 AM
32	The feedback landscape change has on stream flow and infiltration.	2/14/2015 8:33 AM
33	Specific effects of climate change on regional extremes Improving skills of regional climate models and impact analyses	2/14/2015 6:41 AM
34	Lack of knowledge about implications of climate change on variability of local-to-regional scale precipitation patterns	2/13/2015 10:25 PM

Q10 In follow up to Question 9, what specific type of information or assessment do you think is most useful in immediately and positively impacting engineering practices? (Please limit your response to 250 characters)

Answered: 34 Skipped: 10

#	Responses	Date
1	x	5/8/2015 10:08 AM
2	Vulnerability assesment should be the starting point.	5/5/2015 3:15 PM
3	N/A	5/1/2015 9:25 PM
4	monitoring enhancements to soil moisture networks better understanding of seasonal climate outlooks	4/30/2015 4:43 PM
5	Clear, universally adopted methods for determining if the river basin you are studying is displaying non-stationarity. If there is clear non-stationarity, then need a clear, universally-adopted method for performing frequency analyses for that basin.	3/31/2015 4:54 PM
6	vulnerability analyses and extremes scenarios for system analysis	3/23/2015 4:49 PM
7	Water management is local. You need local data to solve local problems.	3/23/2015 10:02 AM
8	This is more a follow-up to Question 8. Engineers need to be comfortable dealing with the demand side of the equation, not just the supply. Risk-based approaches inherently look at that. So, to positive impact engineering practices, we need to get the questions right - how much water is needed when and where (and of what quality). That is more of a social science question. What do people value? What risks are tolerable? What is their elasticity for a certain water resource service?	3/23/2015 9:07 AM
9	social science assessment and application of techniques to assist the public and decision makers in understanding and accepting the risks and options to address the risk	3/23/2015 8:35 AM
10	Incorporating better risk assessment into design, construction & management using resilient and redundant systems concepts.	3/23/2015 8:27 AM
11	Role of no-till on stream flow variability.	3/23/2015 8:19 AM
12	?	3/23/2015 7:44 AM
13	There needs to be objective analysis of trends in mean values and trends in variability. The discussions of change currently rest on model simulations rather than on actual observed change. The understanding of change needs to be based in reality.	3/23/2015 6:49 AM
14	Development of decision tools, high resolution data products	3/17/2015 5:47 PM
15	blended monitoring from in situ and remote platforms, and the further refinement of gridded data fields	3/16/2015 5:56 PM
16	Relationships between land management and soil health.	3/12/2015 11:23 AM
17	Seasonal impacts of precipitation on future water supplies. Most GCM's do a poor job of representing regional climate scenarios at less than an annual basis. This is important because irrigation demand relies on the precipitation distribution during the off-season (Oct-Apr) for soil moisture recharge and within the growing season. Precipitation shortfalls during either of these critical periods increases the demand for irrigation water to offset the loss normal soil moisture recharge. Second, a change in precipitation distributions with in Rocky Mountain region during the "Snow Season" can result in poor reservoir inflows and subsequently reduce available water for irrigation deliveries.	2/25/2015 10:42 AM
18	N/A	2/17/2015 5:08 PM
19	development of widely accepted techniques for accounting for future uncertainty in climate change	2/17/2015 4:48 PM

20	climate predictions need to be downscaled to smaller geographic areas	2/17/2015 3:54 PM
21	There needs to be a stronger relationship between those in the research community and those in the water management community.	2/17/2015 1:21 PM
22	will pipes hold up under lower flushing flow conditions	2/17/2015 1:20 PM
23	More extensive monitoring and analysis of water resources related data, and trends, including precipitation (rain and snowfall), soil moisture, evapotranspiration, ground water, etc.	2/17/2015 12:37 PM
24	Improvement and expansion of in-situ hydroclimate monitoring networks	2/17/2015 12:18 PM
25	don't know	2/17/2015 2:54 AM
26	true costs of doing nothing, and who will pay	2/16/2015 3:02 PM
27	Determine true cost of existing and proposed western infrastructure compared with new infrastructure in the East, i.e. what is the cost vs. the amount of product generated?	2/16/2015 9:15 AM
28	Several reports are coming in regarding water futures in the west considering climate change but the need is great to assess other water basins across the country	2/16/2015 1:48 AM
29	More 360-degree training for engineers to recognize total sets of drivers	2/14/2015 12:34 PM
30	Assessment of real management solutions	2/14/2015 11:31 AM
31	Quality forecasts of changes in variability of extreme events	2/14/2015 10:35 AM
32	How much is reduced tillage impacting groundwater recharge, or reducing stream flows?	2/14/2015 8:33 AM
33	Nonstationary methods for planning and design Scenario approach for planning and design Adaptive risk management methods	2/14/2015 6:41 AM
34	Mesoscale atmospheric models incorporating land-use/land-cover drivers, though these are far from perfect	2/13/2015 10:25 PM

Q11 Do you feel the current curriculum in environmental engineering or in the sciences is adequate to inform a graduate (B.S. degree) of the challenges facing water resource management this century?

Answered: 34 Skipped: 10

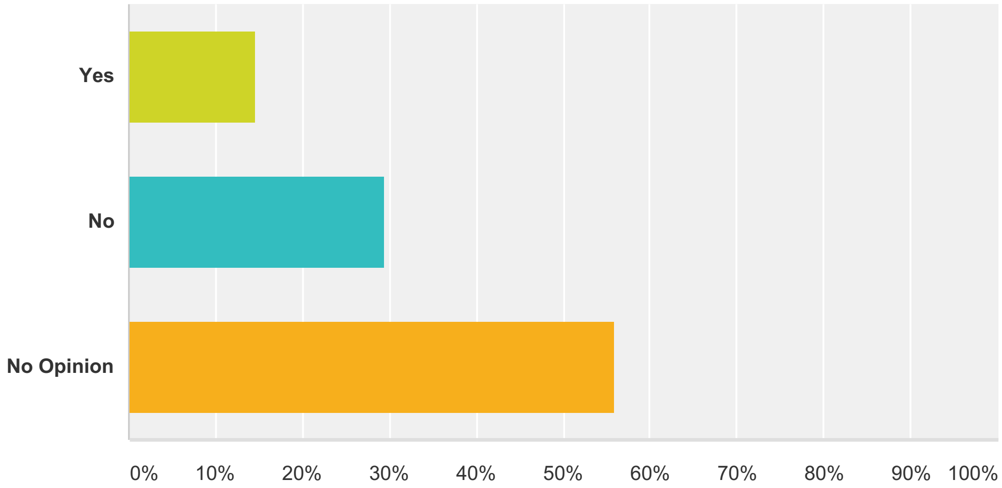

Answer Choices	Responses	
Yes	**14.71%**	5
No	**29.41%**	10
No Opinion	**55.88%**	19
**Total**		**34**

Q12 Please elaborate briefly on the type of curriculum changes needed (e.g. an undergraduate course on thehuman impacts of weather/climate, surface hydrology; water management; hydrometeorology; land management).

Answered: 10 Skipped: 34

#	Responses	Date
1	Engineering is an art. I suggest a broad basic education and then an intern program. Medicine is a good example. You need to learn engineering by doing engineering. The best thing we can do for engineering education is to build a more formal internship program, where budding engineers are paid living wages or better to develop their skills while doing useful work.	3/23/2015 10:05 AM
2	From my minimal interaction with undergrad curricula, it seems more of a focus on the social science aspects of IWRM, and especially of how people see risk. Probably more systems thinking too.	3/23/2015 9:09 AM
3	Helping the engineer understand how to work with the non technical decision makers to address the problem. We know how to fix the problem, the issue is how to support and funding to implement solutions	3/23/2015 8:37 AM
4	The example would be a good add but so would more work to understand the way projects function individually and in systems along with previously built and likely to be built future projects.	3/23/2015 8:32 AM
5	More integrated and holistic links between land - atmosphere exchanges	3/17/2015 5:47 PM
6	more understanding of the implications of engineering on economy, environment, equity	2/16/2015 3:03 PM
7	from my perspective, curricula are light on climate and climate change	2/16/2015 1:49 AM
8	Hard to handle at UG level. Introduce to sustainability there but emphasize more systemic approaches at grad level	2/14/2015 12:35 PM
9	Courses in implementation; I.e., political science and public communications	2/14/2015 11:32 AM
10	Climate change Drivers of change Risk management methods	2/14/2015 6:42 AM

Q13 Which state or region with which you are most concerned with in regards to your profession

Answered: 34 Skipped: 10

#	Responses	Date
1	Ohio Valley	5/8/2015 10:09 AM
2	West and Southwest	5/5/2015 3:16 PM
3	National	5/1/2015 9:26 PM
4	North Central U.S.	4/30/2015 4:44 PM
5	Washington State	3/31/2015 4:55 PM
6	California	3/23/2015 4:50 PM
7	US	3/23/2015 10:07 AM
8	Variable - I work nationally. Areas of particular interest include Hawaii/Asia Pacific, California	3/23/2015 9:11 AM
9	nationwide	3/23/2015 8:37 AM
10	Badly worded question, but CA and FL are obvious concerns with large populations and sensitive water environmental linkages.	3/23/2015 8:36 AM
11	Kansas	3/23/2015 8:19 AM
12	NC	3/23/2015 7:44 AM
13	Middle Atlantic	3/23/2015 6:50 AM
14	midwest and eastern half of the US	3/17/2015 5:48 PM
15	California	3/16/2015 5:56 PM
16	Entire nation	3/12/2015 11:24 AM
17	Nebraska	2/25/2015 10:42 AM
18	California	2/17/2015 5:08 PM
19	Texas	2/17/2015 4:48 PM
20	upper colorado rver basin	2/17/2015 3:54 PM
21	U. S. Midwest	2/17/2015 1:22 PM
22	Colorado	2/17/2015 1:21 PM
23	Seventeen western states and Alaska	2/17/2015 12:38 PM
24	western U.S.	2/17/2015 12:19 PM
25	Southwest	2/17/2015 2:55 AM
26	nationwide	2/16/2015 3:04 PM
27	Alabama	2/16/2015 9:15 AM
28	southwest	2/16/2015 1:50 AM
29	Colorado	2/14/2015 12:35 PM
30	Southwest US	2/14/2015 11:34 AM
31	western US	2/14/2015 10:35 AM

32	Kansas and the HIgh Plains	2/14/2015 8:34 AM
33	Florida	2/14/2015 6:42 AM
34	Mid South	2/13/2015 10:26 PM

Q14 What is your professional affiliation?

Answered: 34 Skipped: 10

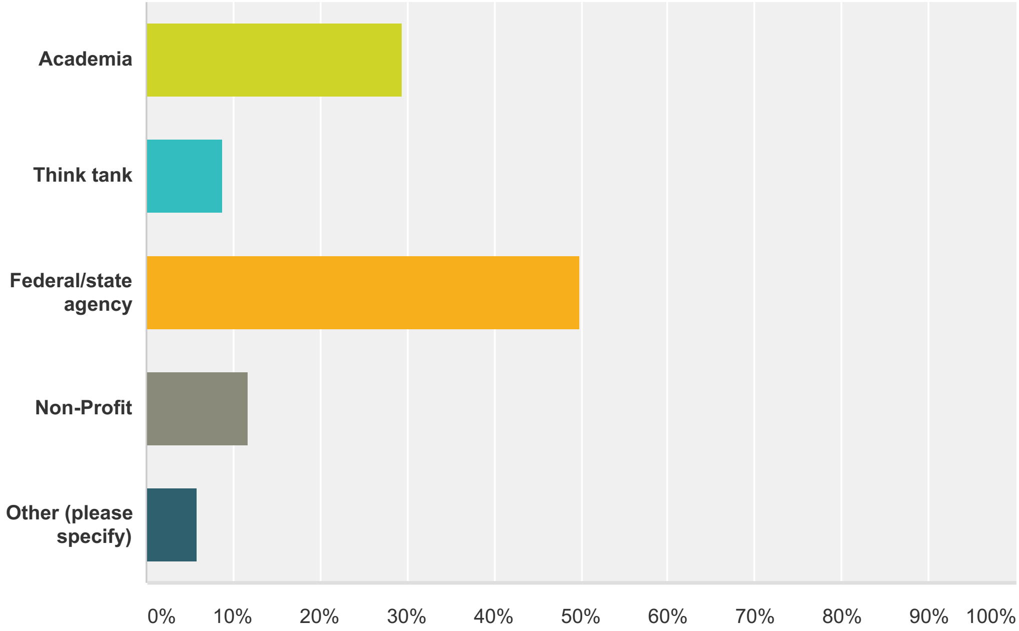

Answer Choices	Responses	
Academia	**29.41%**	10
Think tank	**8.82%**	3
Federal/state agency	**50.00%**	17
Non-Profit	**11.76%**	4
Other (please specify)	**5.88%**	2
**Total Respondents: 34**		

#	Other (please specify)	Date
1	Did you leave out for profit firms on purpose?	3/23/2015 10:07 AM
2	Retired state agency, then consultant, now non-profit	2/14/2015 11:34 AM
